# 16S rRNA gene-based association study identified microbial taxa associated with pork intramuscular fat content in feces and cecum lumen

**DOI:** 10.1186/s12866-017-1055-x

**Published:** 2017-07-19

**Authors:** Shaoming Fang, Xingwei Xiong, Ying Su, Lusheng Huang, Congying Chen

**Affiliations:** State Key Laboratory of Pig Genetic Improvement and Production Technology, Jiangxi Agricultural University, Nanchang, 330045 China

**Keywords:** 16S RNA, IMF content, Gut microbiota, Functional capacity

## Abstract

**Background:**

Intramuscular fat (IMF) that deposits among muscle fibers or within muscle cells is an important meat quality trait in pigs. Previous studies observed the effects of dietary nutrients and additives on improving the pork IMF. Gut microbiome plays an important role in host metabolism and energy harvest. Whether gut microbiota exerts effect on IMF remains unknown.

**Results:**

In this study, we investigated the microbial community structure of 500 samples from porcine cecum and feces using high-throughput 16S rRNA gene sequencing. We found that phylogenetic composition and potential function capacity of microbiome varied between two types of samples. Bacteria wide association study identified 119 OTUs significantly associated with IMF in the two types of samples (FDR < 0.1). Most of the IMF-associated OTUs belong to the bacteria related to polysaccharide degradation and amino acid metabolism (such as *Prevotella*, *Treponema*, *Bacteroides* and *Clostridium*). Potential function capacities related to metabolisms of carbohydrate, energy and amino acids, cell motility, and membrane transport were significantly associated with IMF content. FishTaco analysis suggested that the shifts of potential function capacities of microbiome associated with IMF might be caused by the IMF-associated microbial taxa.

**Conclusions:**

This study firstly evaluated the contribution of gut microbiome to porcine IMF content. The results presented a potential capacity for improving IMF through modulating gut microbiota.

**Electronic supplementary material:**

The online version of this article (doi:10.1186/s12866-017-1055-x) contains supplementary material, which is available to authorized users.

## Background

Mammalian gut microbial community is a heterozygous ecosystem and composed of thousands of microbial species [1]. Gut microbiota influences many important host physiological functions, such as modulation of food intake, metabolism, immune system activation, epithelial cell proliferation and resistance to infection [2]. Some evidences demonstrated that gut microbiota contributes to the development of host fatness. For instances, Backhed et al. showed that germ-free mice have 40% less fat mass than normal conventionalized mice [3]. Turnbaugh et al. confirmed that colonization of germ-free mice with the gut microbiota from obese donors resulted in an increase of total body fat mass [4]. However, some studies have indicated that there is no consistent association between obesity and gut microbiota or only a marginal effect of the microbiome on obesity in humans [5, 6]. Although it is an open-and-shut discussion about the contribution of the gut microbiome to host fatness, further studies have linked some essential gut microbiota-derived metabolites to host metabolic status. Devadder et al. revealed that microbial degradation of dietary fiber to SCFAs plays causal roles in the metabolic benefits [7]. Semova et al. found that metabolites produced by a Firmicutes strain could increase the number of lipid droplets in enterocytes, while metabolites produced by a Bacteroidetes strain or a Proteobacteria strain did not exhibit this effect [8].

Intramuscular fat (IMF) accumulates among muscle fibers or within muscle cells. High IMF in pigs is responsible for “marbling meat”. It is an economically important meat quality trait and contributes to meat sensory properties, such as special flavor, juiciness and tenderness. In humans, Intramuscular fat is associated with human metabolic syndrome [9]. The starting time of IMF, subcutaneous fat (backfat) and visceral fat (abdominal fat) deposition in pigs is different. Visceral fat firstly deposited, followed by subcutaneous fat, and intramuscular fat lastly deposited [10, 11]. Early studies identified several quantitative trait loci (QTL) on pig genome associated with IMF content [12–14]. Nevertheless, most of these QTLs could explain less than 5% of the variation of IMF content. Several studies observed the effects of diet nutrients and additives on improving the pork IMF content [15, 16]. Recent years, the studies have also uncovered the effect of gut microbiota on pig fat deposition. He et al. identified several gut microbial taxa showing significant associations with porcine backfat thickness and abdominal fat weight [17]. Yan et al. reported that transplanting gut microbiota derived from obese pigs to germ-free mice could enhance lipogenesis in the gastrocnemius muscles. Yan et al. suggested that high fat diets induced an improvement of body weight gain, feed efficiency and backfat accumulation in pigs through modulating hindgut microbial community [18]. However, whether gut microbiota plays a role in porcine IMF remains unknown.

The main objective of this study was to identify the microbial taxa and potential function capacity of gut microbiome associated with porcine IMF content with samples from different gut locations. We also identified bacteria and potential function capacities enriched in each of cecum lumen and feces.

## Methods

### Experimental animals and sample collection

Five hundred pigs from two pig populations were used in this study, including 256 Erhualian (EHL) and 244 Bamaxiang (BMX) pigs. All pigs were healthy and had not received antibiotics, probiotics or prebiotics for at least 2 months before sampling. Feeding, management and sampling of experimental pigs were described in detail in our previous publication [17]. In brief, commercial formula diet was provided to pigs two times a day. Water was offered *ad libtium*. Fecal samples from BMX pigs were individually collected at 3 days before the pigs were transported to the slaughterhouse. Cecum lumen samples were harvested from all 256 EHL pigs within 30 min after slaughter at age of 300 ± 3 days. All fecal and lumen samples were immediately dipped into liquid nitrogen for transportation, and then stored at −80 °C until use.

To determine IMF content of experimental pigs, longissimus dorsi muscles were sampled within 30 min after slaughter. All fresh meat samples were dried to constant weight in the oven, and then grinded into powder. Ether was used to extract IMF with the routine Soxhlet extraction method.

### Microbial DNA extraction and 16S rRNA gene sequencing

The detailed procedures of microbial DNA extraction and 16S rRNA gene sequencing were described in our previous publication [17]. Briefly, microbial DNA was extracted using QIAamp DNA Stool Mini Kit (QIAGEN, Germany). The barcoded fusion forward primer 515F (5′-GTGCCAGCMGCCGCGGTAA-3′) and the reverse primer 806R (5′-GGACTACHVGGGTWTCTAAT-3′) were used to amplify the V4 hyper variable region of the 16S rRNA gene. Barcoded V4 amplicons were sequenced using the paired-end method on Illumina MiSeq platform (Illumina, USA) following the standard protocols.

### 16S rRNA gene sequencing data analysis

Firstly, the raw sequencing data were removed the barcodes and low quality sequences to obtain the clean data. High-quality paired-end reads were merged into tags using FLASH (v.1.2.11) [19]. To normalize the sequencing depth, we rarefied the library size of microbial sequences to 10,000 tags per sample before further analysis. USEARCH (v7.0.1090) was used to pick Operational Taxonomic Unit (OTU) at 97% sequence identity [20]. We filtered out those OTUs which had relative abundance <0.05% and were presented in less than 1% of the experimental pigs from further analysis. We performed taxonomic assignments for the aligned sequences using the Ribosomal Database Project (RDP) classifier program (v.2.20) [21]. The abundance and diversity indices were generated using Quantitative Insights Into Microbial Ecology (QIIME v.1.70) [22]. Linear discriminate analysis effect size (LEfSe) was used to identify the bacteria enriched in cecum lumen and feces, respectively [23].

### Association analysis

The residuals of IMF content corrected the effects of sex and batch were used for further association analysis between phenotypic IMF and the relative abundances of bacteria. Because of the non-normal distribution of the relative abundances of OTUs in the tested pigs, association analysis was performed with a two-part model method as described previously [24]. In brief, the two-part model association analysis included both binary and quantitative model. The binary model describes a binomial analysis that tests for association of detecting a microbe with IMF content. The quantitative model tests for association between the abundance of the detected microbes and IMF content. Only the samples where that microbe was identified were included in analysis. Considering the effect of both binary and quantitative features, a meta-analysis was also performed using an unweighted Z method. The final association *P* value was set as the minimum of *P* values of binary, quantitative and meta-analysis. Skewness correction was performed by 1000 × permutation tests. False discovery rate (FDR) < 0.1 was set as the significance threshold.

### Functional prediction of fecal and cecum lumen microbiome

Functional capacity of microbial community was predicted using PICRUSt online Galaxy version [25]. The closed reference OTU table was generated from quality control reads in QIIME (v1.7.0) against the Greengenes database. Normalization, metagenome prediction and function categorization based on KEGG (Kyoto Encyclopedia of Genes and Genomes) pathways were performed by PICRUSt according to a standard analysis process. Comparison of the secondary-class KEGG pathways between cecum and fecal samples was performed using LEfSe. Correlation coefficients between IMF values and relative abundances of KEGG pathways were calculated by using MaAsLin online Galaxy version [26]. The significant threshold was set at FDR < 0.05.

### Identifying the taxonomic drivers of functional shifts

To evaluate the contribution of the IMF-associated OTUs to functional shifts of gut microbiome, we used FishTaco analysis to establish the correlation between IMF-associated OTUs and functional capacities as described by Borenstein et al. [27]. Because FishTaco software has been only used to treat with the dataset from case-control study, we chose those samples whose IMF values were ranked the top or lowest 5% for this analysis. Finally, 26 cecum lumen samples (high IMF: 13, low IMF: 13) and 24 fecal samples (high IMF: 12, low IMF: 12) were used for the analysis. The profiles of the relative abundances of IMF-associated OTUs and predicted function capacities were inputted into FishTaco software. Multi_taxa module was run to assess the taxonomic contribution to functional shifts. The output result was visualized using ggplot2 in R package.

## Results

### Bacteria and potential function capacities enriched in cecum lumen and feces

**Fig. 1:**
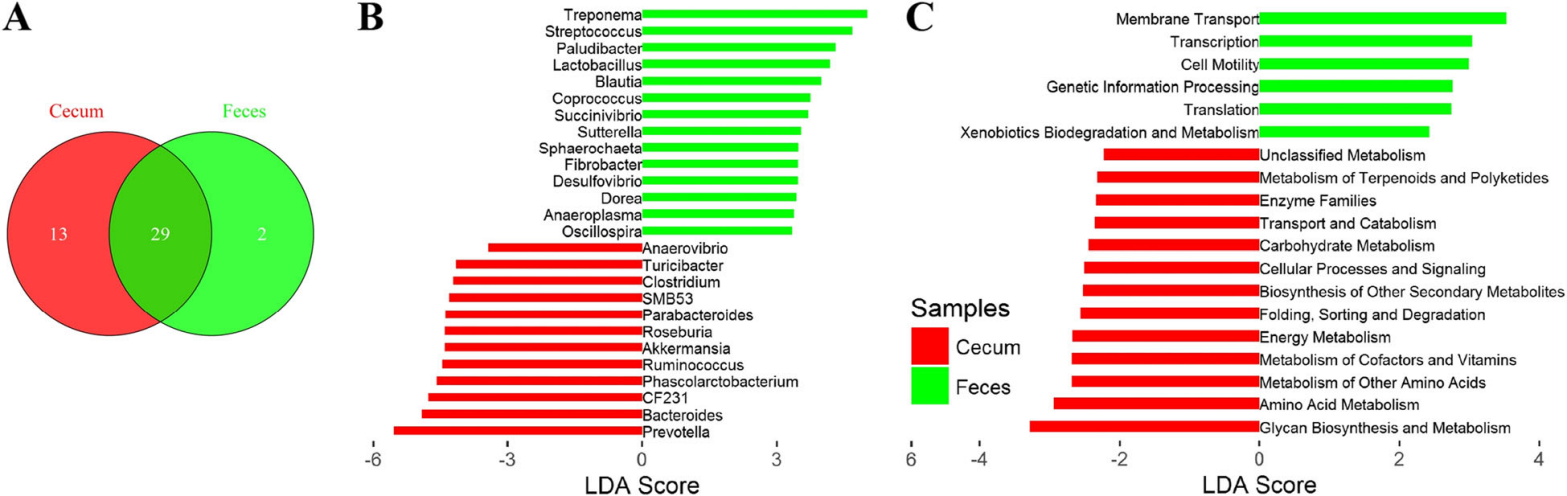
Bacterial taxa and potential function capacities enriched in the cecum and feces. **a** Number of bacterial genera shared between cecum and feces. **b** Comparison of relative abundances of common genera between cecum and feces using LEfSe. **c** Comparison of abundances of KEGG items between cecum lumen and fecal samples using LEfSe

Cecum lumen obtained an average of 718 OTUs in the tested samples, ranging from 195 to 995, while the average number of OTUs for fecal samples was 888, which ranged from 618 to 1066. The α-diversity of microbial community of cecum lumen and feces was described in our previous publication [17]. Here we focused on the identification of bacterial genera enriched in cecum lumen and feces, respectively. A total of 29 genera were identified in both cecum lumen and feces, while 13 genera were specific to cecum and two genera were unique to feces (Fig. 1a). We further performed a LEfSe analysis on the 29 common taxa to compare their relative abundances in the two types of samples. As shown in Fig. 1b, 26 out of the 29 genera showed distinct relative abundances between cecum lumen and feces (*P* < 0.05), including 12 genera enriched in the cecum lumen and 14 genera showing higher abundances in the feces.

To compare the potential function capacity of microbial community between cecum lumen and feces, the relative abundances of KEGG pathways were predicted by PICRUSt based on 16S rRNA gene sequences. We found 39 KEGG pathways shared by both the cecum lumen and fecal samples. LEfse analysis identified 13 pathways enriched in the cecum lumen samples, while six pathways were enriched in the fecal samples (Fig. 1c).

### Microbial taxa associated with IMF content

We identified 119 OTUs which were significantly associated with IMF in the two types of samples using two-part model association analysis (FDR < 0.1). In the cecum lumen samples, 16 OTUs were positively associated with IMF and 38 OTUs had negative correlations with IMF. Among these 54 OTUs, 16 associations (30%) were identified by binary analysis, 14 associations (26%) were detected by quantative analysis, and the other 24 associations (44%) were identified by meta-analysis. Meanwhile, we detected 65 OTUs significantly associated with IMF content in the fecal samples, including 27 OTUs positively associated with IMF and 38 OTUs negatively associated with IMF. Among these 65 IMF-associated OTUs, 19 (29%) were obtained by binary analysis, 22 (34%) by quantative analysis and 24 (37%) by meta-analysis.

**Fig. 2:**
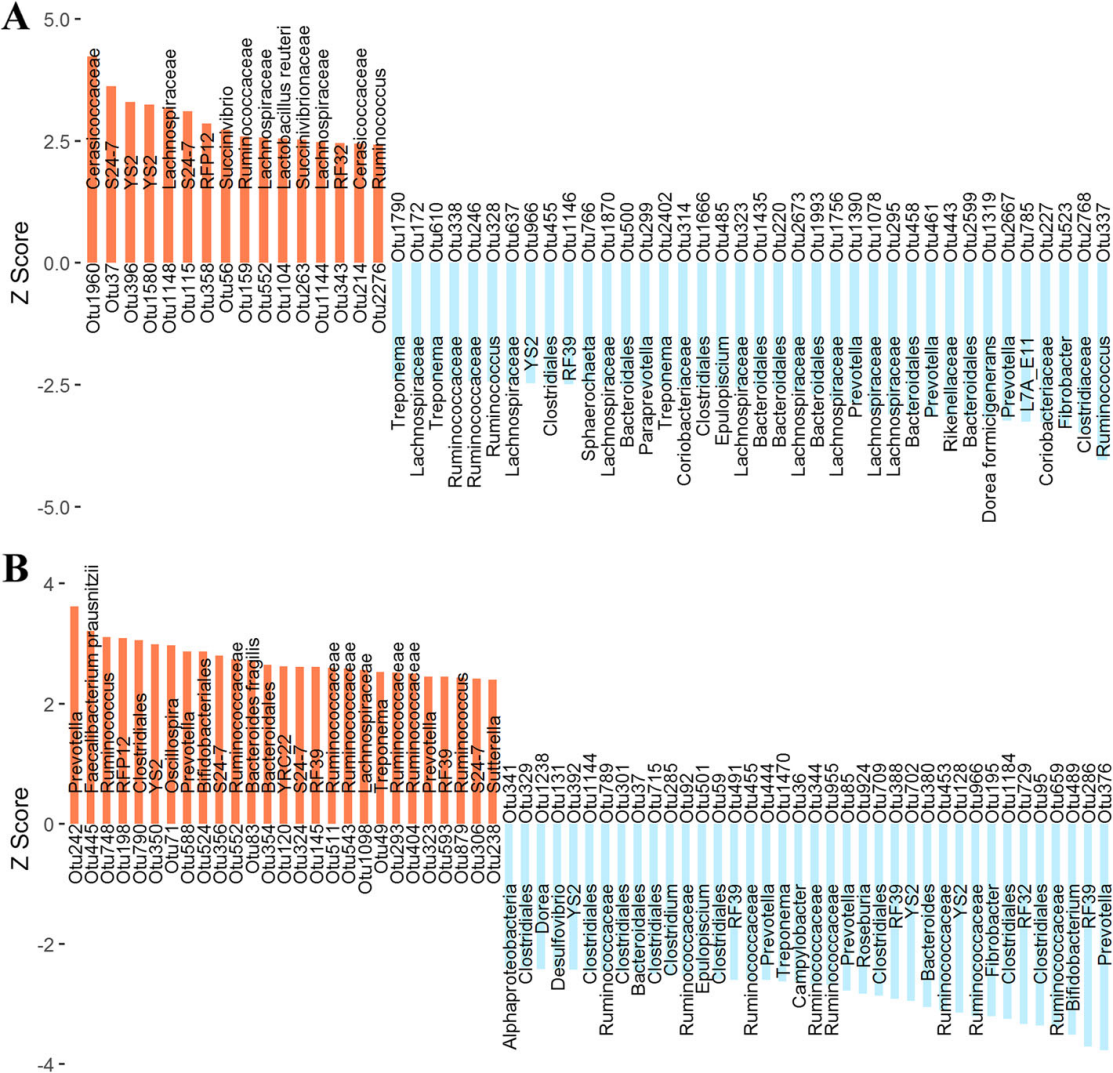
The IMF-associated OTUs. **a** The 54 IMF-associated OTUs identified in the cecum lumen samples (FDR < 0.1) are shown as Z score, the red bar corresponds to positive correlation and the blue bar corresponds to negative correlation, the text on the bar corresponds to microbial taxa annotated to the OTU. **b** The 65 IMF-associated OTUs identified in fecal samples (FDR < 0.1)

We annotated these 119 IMF-associated OTUs to microbial taxa. As shown in Fig. 2, Additional file 1: Table S1 and Additional file 2: Table S2. In the cecum lumen samples, 13 OTUs were annotated to the order level, including six OTUs annotated to *Bacteroidales,* three OTUs to *YS2*, two OTUs to *Clostridiales*, one OTU to each of the *RF32* and *RF39*. Twenty-four OTUs were annotated to the family level, including 11 OTUs annotated to *Lachnospiraceae*, three OTUs to *Ruminococcaceae*, two OTUs to each of *S24–7*, *Cerasicoccaceae* and *Coriobacteriaceae*, and one OTU to each of *Clostridiaceae*, *RFP12*, *Rikenellaceae* and *Succinivibrionaceae*. At the genus level, three OTUs were annotated to each of *Prevotella*, *Treponema* and *Ruminococcus*, and the other seven OTUs to *Epulopiscium*, *Fibrobacter*, *L7A_E1*, *Paraprevotella*, *Rikenellaceae*, *Sphaerochaeta* and *Succinivibrio*. Only two OTUs were annotated to the species level, including *Dorea formicigenerans* and *Lactobacillus reuteri*. In the fecal samples, one OTU was annotated to the class *Alphaproteobacteria*. Twenty-two OTUs were annotated to the order level, including nine OTUs annotated to *Clostridiales*, five OTUs to *RF39*, four OTUs to *YS2*, two OTUs to *Bacteroidales* and one OTU to each of *Bifidobacteriales* and *RF32*. At the family level, 13 OTUs were annotated to *Ruminococcaceae*, three OTUs to *S24–7*, one OTU to each of *Lachnospiraceae* and *RFP12*. Twenty-two OTUs were annotated to the genus level, including six OTUs annotated to *Prevotella*, two OTUs to *Treponema*, two OTUs to *Ruminococcus* and one OTU to each of *Bacteroides*, *Bifidobacterium*, *Campylobacter*, *Clostridium*, *Desulfovibrio*, *Dorea*, *Epulopiscium*, *Fibrobacter*, *Oscillospira*, *Roseburia*, *Sutterella* and *YRC22.* Two IMF-associated OTUs belong to the species *Bacteroides fragilis* and *Faecalibacterium prausnitzii*.

### Association between predicted function capacity of gut microbiome and IMF content

**Fig. 3:**
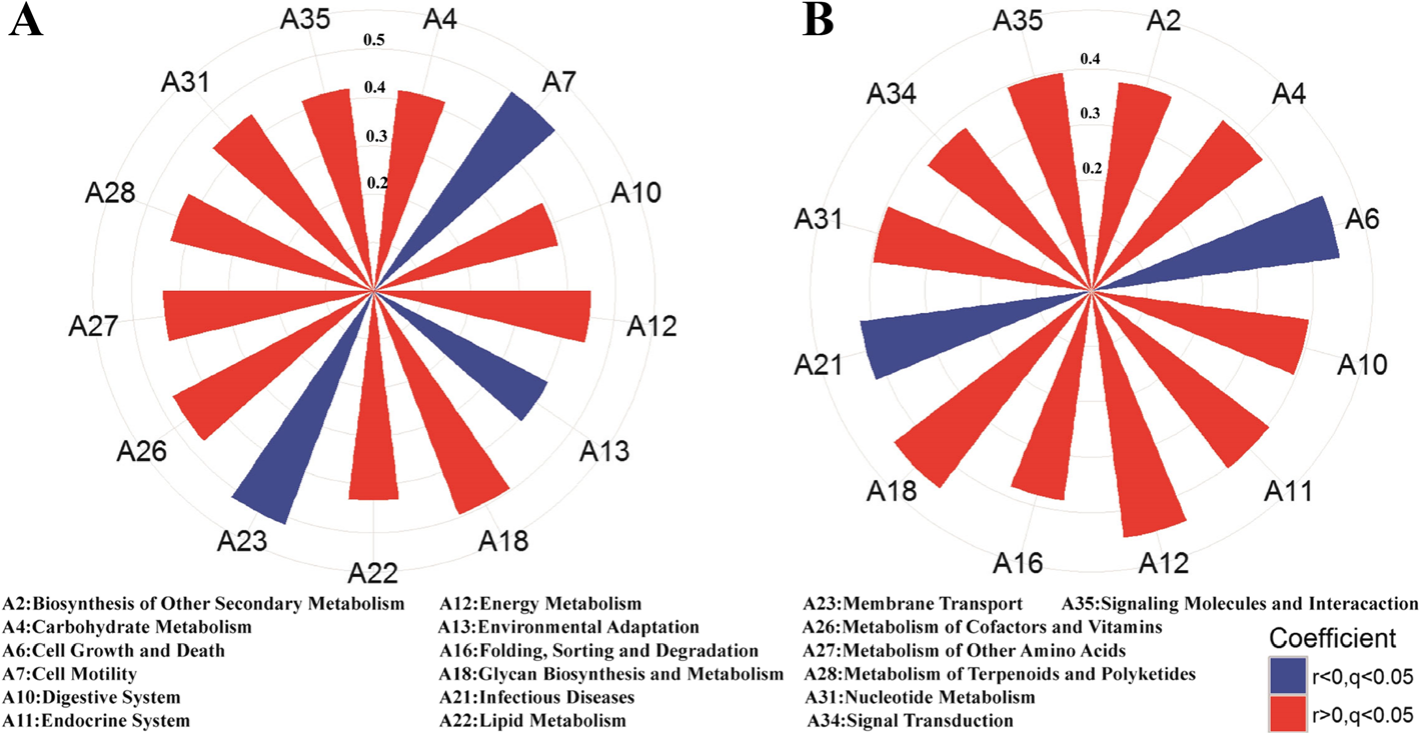
The association between KEGG items and IMF content. **a** KEGG items associated with IMF content in the cecum lumen samples at FDR < 0.05. Values on different cycles represent correlation coefficient. Ax on the edge of the cycle represents the KEGG items. Red sectors represent for positive associations and blue sectors represent for negative associations. **b** KEGG items associated with IMF content in the fecal samples

MaAsLin analysis identified 13 and 12 KEGG pathways related to IMF content in the cecum lumen and stool, respectively. In the cecum lumen samples, digestive system, energy metabolism, glycan biosynthesis and metabolism, lipid metabolism, carbohydrate metabolism, metabolism of cofactors and vitamins, metabolism of other amino acids, metabolism of terpenoids and polyketides, nucleotide metabolism, signaling molecules and interaction had positive correlations with IMF content, while cell motility, environmental adaptation, and membrane transport showed negative correlations with IMF content (Fig. 3a). In the fecal samples, biosynthesis of other secondary metabolites, carbohydrate metabolism, digestive system, endocrine system, energy metabolism, glycan biosynthesis and metabolism, signal transduction, and signaling molecules and interaction were positively associated with IMF content, while cell growth and death, and infectious diseases were negatively associated with IMF content (Fig. 3b). Carbohydrate metabolism, digestive system, energy metabolism, glycan biosynthesis and metabolism, nucleotide metabolism, and signaling molecules and interaction shared the positive association with IMF in both cecum lumen and feces samples.

### Contribution of the IMF-associated OTUs to functional shifts of microbial community

**Fig. 4:**
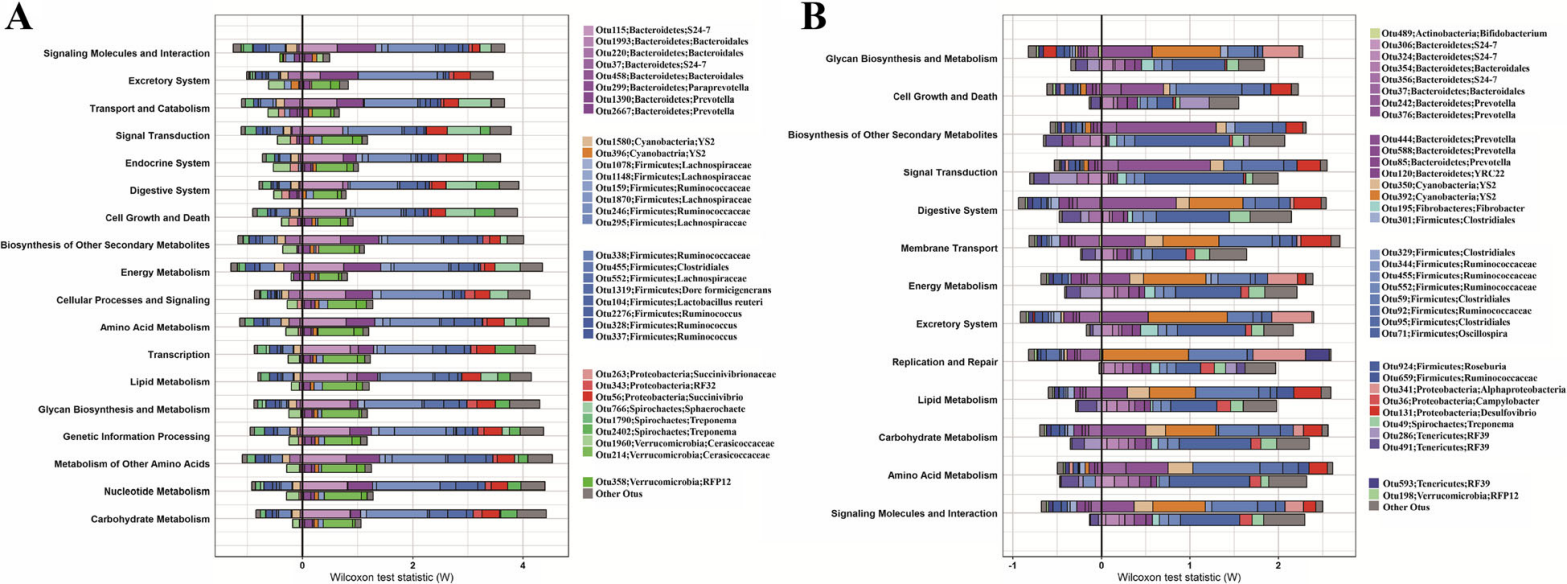
The contribution of IMF-associated OTUs to the function shifts of IMF-associated KEGG pathways. For each pathway, the sum of OTU contribution scores matches the observed OTU-based functional shift score. For each function term, the bar on the top-right of Y axis represents high IMF-associated OTUs driving functional shift; the bar on the top-left of Y axis indicates high IMF-associated OTUs attenuating functional shift; the bar on the bottom-right of Y axis represents low IMF-associated OTUs driving functional shift; the bar on the bottom-left of Y axis shows low IMF-associated OTUs attenuating functional shift. **a** Cecum lumen samples. **b** Fecal samples

We estimated the contribution of IMF-associated OTUs to functional shifts of microbial community of cecum lumen and feces. As shown in Fig. 4a, OTU246 (*Ruminococcaceae*), OTU115 (*Bacteroidetes, S24–7*), OTU214 (*Cerasicoccaceae*), OTU552 (*Lachnospiraceae*), OTU1319 (*Dorea formicigenerans*), OTU1390 (*Prevotella*) and OTU2402 (*Treponema*) in the cecum lumen samples mostly contributed to the function capacities associated with IMF, especially, to the metabolism-associated function terms. The similar result was obtained in the fecal samples where OTU71 (*Oscillospira*), OTU59 (*Clostridiales*), OTU376 (*Prevotella*), OTU392 (*YS2*), OTU350 (*YS2*) and OTU131 (*Desulfovibrio*) had the largest contributions to the IMF-associated function capacities (Fig. 4b). This result suggested that IMF-associated function shift of gut microbiome might be caused by IMF-associated OTUs.

## Discussion

In this study, we identified a total of 119 OTUs and 25 KEGG pathways related to IMF content in porcine cecum lumens and stools. To our knowledge, this is the first study systematically evaluating the association of gut microbiome with porcine IMF content from both bacteria and potential functional capacity of microbial community.

We identified a significantly larger number of cecum lumen-specific bacteria. This was consistent with the report that cecum and colon have high richness and diversity of microbiota [28]. The potential function capacity of microbial community was also distinctly different between cecum lumen and stool (Fig. 1c). Metabolism-related function terms, such as carbohydrate metabolism, amino acid metabolism, energy metabolism, metabolism of cofactors and vitamins, and Glycan biosynthesis and metabolism were significantly enriched in the cecum lumen. As we have well known, cecum is the main gut location where bacteria ferment polysaccharide to provide energy, and synthesize essential amino acids and vitamins [29, 30].

To our best knowledge, for the first time, cecum lumen and stool samples were used to evaluate the association between gut microbiome and IMF. We used a powerful two-part model method to establish the association of gut microbial taxa with IMF content [24]. This approach accounted for both binary and quantitative features of microbial data to maximize the statistic power. Furthermore, it overcame the problem of the non-normal distribution of OTU abundance to make the results more accurate. Although similar numbers of IMF-associated OTUs were identified in both types of samples (54 vs. 65), most members of the IMF-associated OTUs in the cecum lumen belong to *Lachnospiraceae*, while the IMF-associated OTUs in the feces were mainly annotated to *Ruminococcaceae* (Fig. 2 and Additional file 1: Table S1 and Additional file 2: Table S2). This was consistent with the distinct phylogenetic compositions between cecum lumen and feces.

Previous studies indicated that the expression level of host genes involved in glucose and amino acid metabolism or dietary intervention (low-protein and high-glucose) could affect porcine IMF deposition [31–34]. Coincidentally, we found that most of the IMF-associated bacteria identified in the cecum lumen and fecal samples have been reported to play important roles in the metabolisms of dietary nondigestible polysaccharide and amino acid. For examples, *Lachnospiraceae*, *Ruminococcaceae*, *Prevotella* and *Treponema* ferment dietary complex polysaccharide to produce short chain fatty acids (SCFAs) and have been reported to affect host inflammation and obesity in humans [35–39]. Members of *Bacteroidales* (e.g. *Bacteroides*) and *Clostridiales* (e.g. *Clostridia* and *Clostridium*) appear to be prevalent species involved in amino acid fermentation [40]. Members of *Clostridiales* produce branched-chain amino acids (leucine, isoleucine and valine) through amino acid catabolism, which was associated with obesity and inflammation [41–43]. In our previous studies, we also identified that the above bacterial taxa were significantly associated with porcine fatness traits, suggesting that bacteria involving in digesting polysaccharide and amino acid, and SCFAs producing by polysaccharide fermentation were related to the deposition of IMF, abdominal fat and backfat [17, 44]. However, we also identified several bacteria specifically associated with IMF, such as *Coriobacteriaceae*, *Bifidobacterium*, *Roseburia, Lactobacillus reuteri* and *Faecalibacterium prausnitzii*. These bacteria have been reported to be involved in obesity in humans and mice, e.g. *Coriobacteriaceae* was associated with host dyslipidemic phenotypes in mice and humans in the context of obesity, metabolic syndrome and hypercholesterolemia [45–47]. *Bifidobacterium* ameliorates visceral fat accumulation and insulin sensitivity in HF-diet-fed rats [48, 49]. *Roseburia* plays an important role in producing butyrate from dietary carbohydrates and anti-inflammatory properties [50, 51]. And *Lactobacillus reuteri* could improve incretion and insulin secretion in glucose-tolerant humans [52].

Functional prediction of microbial community with 16S rRNA gene sequencing data and FishTaco analysis constructed the bridge linking potential function capacity of microbome with IMF-associated bacterial taxa. FishTaco analysis suggested that functional shifts of gut microbiome related to IMF might be caused by IMF-associated microbial taxa. Carbohydrate metabolism, energy metabolism and the metabolism of other amino had positive association with IMF content. This was consistent with the result that IMF-associated bacteria were associated with digestion of dietary polysaccharide and amino acids (described above). The function terms of cell motility, membrane transport, and environmental adaptation had negative associations with IMF content. Interestingly, previous studies indicated that cell motility, membrane transport and oxidative stress resistance were associated with type 2 diabetes and obesity [53, 54].

## Conclusions

In this study, we identified 119 OTUs that were significantly associated with IMF content. Most of the IMF-associated OTUs belong to the bacteria involved in polysaccharides and amino acid metabolism. Potential function capacities related to metabolisms of carbohydrate, energy and amino acids were significantly associated with IMF content. Functional shifts of gut microbiome related to IMF should be caused by the IMF-associated microbial taxa. The results from this study gave a comprehensive knowledge about the contribution of gut microbiome to porcine IMF content, and presented a potential capacity for improving IMF through modulating gut microbiota.

## Additional files

Additional file 1: Table S1. OTUs significantly associated with IMF content (FDR < 0.1) in the cecum lumen samples. (XLSX 13 kb)

Additional file 2: Table S2. OTUs significantly associated with IMF content (FDR < 0.1) in the fecal samples. (XLSX 13 kb)

## Abbreviations

IMF: Intramuscular fat
KEGG: Kyoto Encyclopedia of Genes and Genomes
OTU: Operational Taxonomic Unit
PCoA: Principal Coordinate Analysis
rRNA: Ribosomal Robonucleic Acid

## References

[1] Kim HB, Isaacson RE (2015). The pig gut microbial diversity: understanding the pig gut microbial ecology through the next generation high throughput sequencing. Vet Microbiol.

[2] Flint HJ, Scott KP, Louis P, Duncan SH (2012). The role of the gut microbiota in nutrition and health. Nat Rev Gastroenterol Hepatol.

[3] Bäckhed F, Ding H, Wang T, Hooper LV, Gou YK, Nagy A, Semenkovich CF, Gordon JI (2004). The gut microbiota as an environmental factor that regulates fat storage. Proc. Natl. Acad. Sci. U.S.A.

[4] Turnbaugh PJ, Ley RE, Mahowald MA, Magrini V, Mardis ER, Gordon JI (2006). An obesity-associated gut microbiome with increased capacity for energy harvest. Nature.

[5] Finucane MM, Sharpton TJ, Laurent TJ, Pollard KS (2014). A taxonomic signature of obesity in the microbiome? Getting to the guts of the matter. PLoS One.

[6] Sze MA, Schloss PD (2016). Looking for a signal in the noise: revisiting obesity and the Microbiome. MBio.

[7] De Vadder F, Kovatcheva-Datchary P, Goncalves D, Vinera J, Zitoun C, Duchampt A, Backhed F, Mithieux G (2014). Microbiota-generated metabolites promote metabolic benefits via gut-brain neural circuits. Cell.

[8] Semova I, Carten JD, Stombaugh J, Mackey LC, Knight R, Farber SA, Rawls JF (2012). Microbiota regulate intestinal absorption and metabolism of fatty acids in the zebrafish. Cell Host Microbe.

[9] Therkelsen KE, Pedley A, Speliotes EK, Massaro JM, Murabito J, Hoffmann U, Fox CS (2013). Intramuscular fat and associations with metabolic risk factors in the Framingham heart study. Arterioscler Thromb Vasc Biol.

[10] Kouba M, Bonneau M, Noblet J (1999). Relative development of subcutaneous, intermuscular, and kidney fat in growing pigs with different body compositions. J Anim Sci.

[11] Hauser N, Mourot J, De Clercq L, Genart C, Remacle C (1997). The cellularity of developing adipose tissues in Pietrain and Meishan pigs. Reprod Nutr Dev.

[12] de Koning DJ, Janss LL, Rattink AP, van Oers PA, de Vries BJ, Groenen MA, van der Poel JJ, de Groot PN, Brascamp EW, van Arendonk JA (1999). Detection of quantitative trait loci for backfat thickness and intramuscular fat content in pigs (*Sus scrofa*). Genetics.

[13] Sato S, Ohnishi C, Kikuchi T, Kohira K, Egawa S, Terai S, Nakamura T, Arata S, Komatsuda A, Uemoto Y (2014). Evaluation of quantitative trait loci affecting intramuscular fat and reproductive traits in pigs using marker-assisted introgression. Anim Genet.

[14] Sanchez MP, Iannuccelli N, Basso B, Bidanel JP, Billon Y, Gandemer G, Gilbert H, Larzul C, Legault C, Riquet J (2007). Identification of QTL with effects on intramuscular fat content and fatty acid composition in a Duroc x large white cross. BMC Genet.

[15] Cordero G, Isabel B, Menoyo D, Daza A, Morales J, Pineiro C, Lopez-Bote CJ (2010). Dietary CLA alters intramuscular fat and fatty acid composition of pig skeletal muscle and subcutaneous adipose tissue. Meat Sci.

[16] Ramsay TG, Evock-Clover CM, Steele NC, Azain MJ (2001). Dietary conjugated linoleic acid alters fatty acid composition of pig skeletal muscle and fat. J Anim Sci.

[17] He M, Fang S, Huang X, Zhao Y, Ke S, Yang H, Li Z, Gao J, Chen C, Huang L (2016). Evaluating the contribution of gut Microbiota to the variation of porcine fatness with the cecum and fecal samples. Front Microbiol.

[18] Yan H, Potu R, Lu H, Vezzoni DAV, Stewart T, Ragland D, Armstrong A, Adeola O, Nakatsu CH, Ajuwon KM (2013). Dietary fat content and fiber type modulate hind gut microbial community and metabolic markers in the pig. PLoS One.

[19] Magoc T, Salzberg SL (2011). FLASH: fast length adjustment of short reads to improve genome assemblies. Bioinformatics.

[20] Edgar RC (2010). Search and clustering orders of magnitude faster than BLAST. Bioinformatics.

[21] Wang Q, Garrity GM, Tiedje JM, Cole JR (2007). Naive Bayesian classifier for rapid assignment of rRNA sequences into the new bacterial taxonomy. Appl Environ Microbiol.

[22] Caporaso JG, Kuczynski J, Stombaugh J, Bittinger K, Bushman FD, Costello EK, Fierer N, Pena AG, Goodrich JK, Gordon JI (2010). QIIME allows analysis of high-throughput community sequencing data. Nat Methods.

[23] Segata N, Izard J, Waldron L, Gevers D, Miropolsky L, Garrett WS, Huttenhower C (2011). Metagenomic biomarker discovery and explanation. Genome Biol.

[24] Fu J, Bonder MJ, Cenit MC, Tigchelaar EF, Maatman A, Dekens JA, Brandsma E, Marczynska J, Imhann F, Weersma RK (2015). The gut Microbiome contributes to a substantial proportion of the variation in blood Lipids. Circ Res.

[25] Langille MG, Zaneveld J, Caporaso JG, McDonald D, Knights D, Reyes JA, Clemente JC, Burkepile DE, Vega TR, Knight R (2013). Predictive functional profiling of microbial communities using 16S rRNA marker gene sequences. Nat Biotechnol.

[26] Morgan XC, Huttenhower C (2014). Meta'omic analytic techniques for studying the intestinal microbiome. Gastroenterology.

[27] Manor O, Borenstein E (2017). Systematic characterization and analysis of the taxonomic drivers of functional shifts in the human Microbiome. Cell Host Microbe.

[28] Isaacson R, Kim HB (2012). The intestinal microbiome of the pig. Anim Health Res Rev.

[29] Stevens CE, Hume ID (1998). Contributions of microbes in vertebrate gastrointestinal tract to production and conservation of nutrients. Physiol Rev.

[30] Cummings JH, Macfarlane GT (1997). Role of intestinal bacteria in nutrient metabolism. JPEN J Parenter Enteral Nutr.

[31] Liang Y, Yang XM, Gu YR, Tao X, Zhong ZZ, Gong JJ, Chen XH, Lv XB (2015). Developmental changes in the expression of the GLUT2 and GLUT4 genes in the longissimus dorsi muscle of Yorkshire and Tibetan pigs. Genet Mol Res.

[32] Liu JB, Cai X, Xiong H, Zhang HF. Effects of feeding frequency on meat quality traits and Longissimus muscle proteome in finishing pigs. J Anim Physiol Anim Nutr (Berl). 2017;7. doi:10.1111/jpn.12636 10.1111/jpn.12636 28063249

[33] Duan Y, Duan Y, Li F, Li Y, Guo Q, Ji Y, Tan B, Li T, Yin Y (2016). Effects of supplementation with branched-chain amino acids to low-protein diets on expression of genes related to lipid metabolism in skeletal muscle of growing pigs. Amino Acids.

[34] Fisher KD, Scheffler TL, Kasten SC, Reinholt BM, van Eyk GR, Escobar J, Scheffler JM, Gerrard DE (2013). Energy dense, protein restricted diet increases adiposity and perturbs metabolism in young, genetically lean pigs. PLoS One.

[35] Kovatcheva-Datchary P, Nilsson A, Akrami R, Lee YS, De Vadder F, Arora T, Hallen A, Martens E, Bjorck I, Backhed F (2015). Dietary fiber-induced improvement in glucose metabolism is associated with increased abundance of Prevotella. Cell Metab.

[36] De Filippo C, Cavalieri D, Di Paola M, Ramazzotti M, Poullet JB, Massart S, Collini S, Pieraccini G, Lionetti P (2010). Impact of diet in shaping gut microbiota revealed by a comparative study in children from Europe and rural Africa. Proc. Natl. Acad. Sci. U.S.A.

[37] Li S, Yingyi G, Chen L, Lijuan G, Ou S, Peng X (2015). Lean rats gained more body weight from a high-fructooligosaccharide diet. Food Funct.

[38] Duncan SH, Belenguer A, Holtrop G, Johnstone AM, Flint HJ, Lobley GE (2007). Reduced dietary intake of carbohydrates by obese subjects results in decreased concentrations of butyrate and butyrate-producing bacteria in feces. Appl Environ Microbiol.

[39] Biddle A, Stewart L, Blanchard J, Leschine S (2013). Untangling the Genetic basis of Fibrolytic specialization by Lachnospiraceae and Ruminococcaceae in diverse gut communities. Diversity.

[40] Dai ZL, Wu G, Zhu WY (2011). Amino acid metabolism in intestinal bacteria: links between gut ecology and host health. Front Biosci (Landmark Ed).

[41] Ishioka M, Miura K, Minami S, Shimura Y, Ohnishi H (2017). Altered gut Microbiota composition and immune response in experimental Steatohepatitis mouse models. Dig Dis Sci.

[42] Barker HA (1981). Amino acid degradation by anaerobic bacteria. Annu Rev Biochem.

[43] Pataky Z, Genton L, Spahr L, Lazarevic V, Terraz S, Gaia N, Rubbia-Brandt L, Golay A, Schrenzel J, Pichard C (2016). Impact of Hypocaloric Hyperproteic diet on gut Microbiota in overweight or obese patients with nonalcoholic fatty liver disease: a pilot study. Dig Dis Sci.

[44] Yang H, Huang X, Fang S, Xin W, Huang L, Chen C (2016). Uncovering the composition of microbial community structure and metagenomics among three gut locations in pigs with distinct fatness. Sci Rep.

[45] Spencer MD, Hamp TJ, Reid RW, Fischer LM, Zeisel SH, Fodor AA (2011). Association between composition of the human gastrointestinal microbiome and development of fatty liver with choline deficiency. Gastroenterology.

[46] Martinez I, Wallace G, Zhang C, Legge R, Benson AK, Carr TP, Moriyama EN, Walter J (2009). Diet-induced metabolic improvements in a hamster model of hypercholesterolemia are strongly linked to alterations of the gut microbiota. Appl Environ Microbiol.

[47] Zhang C, Zhang M, Wang S, Han R, Cao Y, Hua W, Mao Y, Zhang X, Pang X, Wei C (2010). Interactions between gut microbiota, host genetics and diet relevant to development of metabolic syndromes in mice. ISME J.

[48] An HM, Park SY, Lee DK, Kim JR, Cha MK, Lee SW, Lim HT, Kim KJ, Ha NJ (2011). Antiobesity and lipid-lowering effects of Bifidobacterium spp. in high fat diet-induced obese rats. Lipids Health Dis.

[49] Chen J, Wang R, Li XF, Wang RL (2012). Bifidobacterium adolescentis supplementation ameliorates visceral fat accumulation and insulin sensitivity in an experimental model of the metabolic syndrome. Br J Nutr.

[50] Sheridan PO, Martin JC, Lawley TD, Browne HP, Harris HM, Bernalier-Donadille A, Duncan SH, O'Toole PW, Scott KP, Flint HJ (2016). Polysaccharide utilization loci and nutritional specialization in a dominant group of butyrate-producing human colonic Firmicutes. Microb Genom.

[51] Takahashi K, Nishida A, Fujimoto T, Fujii M, Shioya M, Imaeda H, Inatomi O, Bamba S, Sugimoto M, Andoh A (2016). Reduced abundance of butyrate-producing bacteria species in the fecal microbial Community in Crohn's disease. Digestion.

[52] Simon MC, Strassburger K, Nowotny B, Kolb H, Nowotny P, Burkart V, Zivehe F, Hwang JH, Stehle P, Pacini G (2015). Intake of Lactobacillus reuteri improves incretin and insulin secretion in glucose-tolerant humans: a proof of concept. Diabetes Care.

[53] Qin J, Li Y, Cai Z, Li S, Zhu J, Zhang F, Liang S, Zhang W, Guan Y, Shen D (2012). A metagenome-wide association study of gut microbiota in type 2 diabetes. Nature.

[54] Greenblum S, Turnbaugh PJ, Borenstein E (2012). Metagenomic systems biology of the human gut microbiome reveals topological shifts associated with obesity and inflammatory bowel disease. Proc. Natl. Acad. Sci. U.S.A.

